# Topical Curcumin-Based Cream Is Equivalent to Dietary Curcumin in a Skin Cancer Model

**DOI:** 10.1155/2012/147863

**Published:** 2012-12-13

**Authors:** Kunal Sonavane, Jeffrey Phillips, Oleksandr Ekshyyan, Tara Moore-Medlin, Jennifer Roberts Gill, Xiaohua Rong, Raghunatha Reddy Lakshmaiah, Fleurette Abreo, Douglas Boudreaux, John L. Clifford, Cherie-Ann O. Nathan

**Affiliations:** ^1^Department of Otolaryngology-Head and Neck Surgery, Louisiana State University Health Sciences Center, Shreveport, LA 71130-3932, USA; ^2^Feist-Weiller Cancer Center, Louisiana State University Health Sciences Center, Shreveport, LA 71130-3932, USA; ^3^Department of Biochemistry, Louisiana State University Health Sciences Center, Shreveport, LA 71130-3932, USA; ^4^Department of Pathology, Louisiana State University Health Sciences Center, Shreveport, LA 71130-3932, USA; ^5^Boudreaux's Compounding Pharmacy, Shreveport, LA 71130-3932, USA; ^6^Department of Surgery, Overton Brooks VA Medical Center, Shreveport, LA 71130-3932, USA

## Abstract

Skin squamous cell carcinoma (SCC), the most common cancer in the USA, is a growing problem with the use of tanning booths causing sun-damaged skin. Antiproliferative effects of curcumin were demonstrated in an aggressive skin cancer cell line SRB12-p9 (*P* < 0.05 compared to control). Topical formulation was as effective as oral curcumin at suppressing tumor growth in a mouse skin cancer model. Curcumin at 15 mg administered by oral, topical, or combined formulation significantly reduced tumor growth compared to control (*P* = 0.004). Inhibition of pAKT, pS6, p-4EBP1, pSTAT3, and pERK1/2 was noted in SRB12-p9 cells post-curcumin treatment compared to control (*P* < 0.05). Inhibition of pSTAT3 and pERK1/2 was also noted in curcumin-treated groups *in vivo*. IHC analysis revealed human tumor specimens that expressed significantly more activated pERK (*P* = 0.006) and pS6 (*P* < 0.0001) than normal skin samples. This is the first study to compare topical curcumin to oral curcumin. Our data supports the use of curcumin as a chemopreventive for skin SCC where condemned skin is a significant problem. Prevention strategies offer the best hope of future health care costs in a disease that is increasing in incidence due to increased sun exposure.

## 1. Introduction

The American Cancer Society estimates that 1–1.3 million cases of nonmelanoma skin cancer (NMSC) will be detected annually. Cutaneous SCC accounts for nearly 20% of all skin cancers, and excluding melanoma, 75% of all deaths attributed to skin cancers [1]. Unlike the more prevalent basal cell carcinoma (BCC), SCC is an aggressive tumor that metastasizes with a frequency as high as 12.5% [2]. Prevalence is common in fair complexion Caucasians with lower reported rates in individuals with darker complexions including Asians and Africans. Cutaneous SCC of the face often metastasizes to parotid lymph nodes, which can be detrimental to the facial nerve during treatment and nodes in the neck, as the head and neck are rich in lymphatic networks. Treatment for NMSC may include cryotherapy, electrosurgery, topical 5-fluorouracil, photodynamic therapy, imiquimod, and radiation therapy; however, surgical intervention is the primary treatment modality. When treated early, the five-year cure rate is greater than 90% [3]. NMSC recurrence varies from 8–16%, second lesion recurrence rates are as high as 75% within the first two years and 95% within five years [3]. This suggests a window of opportunity for chemopreventive agents to delay or prevent a recurrence or metastatic spread. Lymph node metastasis in NMSC varies from 0.1 to 28%, with a resulting mortality from 50–75% [4]. Overall five-year survival rates for regional lymph node metastasis are 25–35% [3, 5–7] and less than 20% at ten years [1]. Early cancer detection offers the best window of opportunity for treatment. Early stage skin cancer has a high cure rate, whereas advanced stage cutaneous SCC often develops resistance to chemotherapy. Therefore, research has focused on developing these novel chemopreventive agents to delay or prevent cutaneous SCC formation.

Curcumin, an extract from the Indian spice turmeric, has been investigated in a variety of human cancers including pancreatic, prostate, breast, and head and neck cancer. The first published report demonstrating the topical use of curcumin in cancer reported a sustainable reduction in lesion size and pain [8]. Curcumin has antioxidant, anti-inflammatory, antiangiogenic and anticarcinogenic activity, although its clinical use is limited by low bioavailability [9].

More recently, several studies have examined curcumin's effect in inhibiting skin carcinogenesis. Additionally, numerous reports have identified signaling pathways related to epidermal growth factor receptor (EGFR) that are essential to formation and progression of cutaneous malignancy. The MTOR and MEK/ERK signaling cascades are two of the most well-studied pathways [10]. In a prior study by our group [11] we subcutaneously injected immunodeficient mice with SRB12-p9 skin SCC and demonstrated that curcumin administered by oral gavage significantly inhibited tumor growth and downregulated pS6, a well-established downstream biomarker of the MTOR and MEK/ERK pathways. Curcumin's anti-carcinogenic effects have been linked to inhibition of the MEK/ERK signaling pathway in breast carcinogenesis, and researchers continue to explore these potential biomarkers in other cancers [12]. However, ERKs activity in cutaneous malignancy is not well defined in the literature. Hence, we wanted to determine if topical curcumin was as efficacious as oral curcumin in a SCC skin xenograft model and elucidate the pathways downregulated by curcumin as potential biomarkers for future chemopreventive studies with our topical curcumin cream. In addition, we wanted to observe the potentially additive effects of topical application and oral dosing. We also wished to explore whether the MEK/ERK pathway is overexpressed in human cutaneous SCC and BCC in the hope of identifying a novel intracellular target at which curcumin may act to inhibit tumorigenesis. We hypothesized that pERK and its downstream target pS6 would be overexpressed in cutaneous skin cancers given its role in promoting cellular proliferation in aggressive malignancy. Identifying intermediate endpoints is necessary to assess intervention results for primary cancer prevention and address problems with feasibility posed by large patient numbers, length of study, and cost when cancer occurrence or recurrence is an endpoint [13].

## 2. Materials and Methods

### 2.1. Curcumin

Curcumin C3 Complex (>98% pure) was obtained from Sabinsa Corp. *In vivo* studies were conducted with curcumin (15 mg) suspended in vehicle (100 *μ*L corn oil) for oral gavage feeding or suspended in a vanishing cream paste (15 mg/100 *μ*L cream) for topical administration provided by our study compounding pharmacist (DB).

### 2.2. Cell Lines and Xenografts

The human skin SCC cell line SRB12-p9 was derived by single-cell cloning from aggressive skin SCC SRB12 cells (a gift from Dr. Reuben Lotan, Department of Thoracic Head and Neck Medical Oncology, University of Texas M.D. Anderson Cancer Center in 2003) and was cultured as described [14]. This cell line was chosen due to its sensitivity to curcumin as evidenced in cell culture studies. DNA was isolated from the cell lines using a commercially available DNA purification kit (Qiagen). DNA sample was sent to Genetica (Cincinnati, OH, USA), and the cell line was validated by DNA profiling.

### 2.3. Cell Proliferation

2,000 SRB12-p9 cells per well were seeded in triplicate onto 96 well plates in complete media at 37°C with 5% CO_2_. After adherence, cells were treated with curcumin (0–40 *μ*M) for 0–72 hours. Cell viability was measured using MTS (Promega).

### 2.4. Subcutaneous HNSCC Xenograft Model

Studies were conducted in accordance with the Declaration of Helsinki (1964) and in compliance with Louisiana State University Health Sciences Center Institutional Animal Care and Use Committee guidelines. Animals housed in a barrier facility were maintained on a normal diet *ad lib*. Forty 6–8-week-old Severe Combined Immunodeficiency (SCID) mice were shaved and pretreated with either 0 mg (corn oil), 15 mg curcumin by oral gavage, 15 mg curcumin topical paste, or combined 15 mg oral gavage and 15 mg curcumin topical paste once daily for 3 days prior to squamous cell carcinoma xenograft injection (*n* = 10 per group). Mice were then injected subcutaneously with 1 × 10^6^ SRB12-p9 cells suspended in sterile PBS (Day 0). All mice continued daily treatment with either 0 mg or 15 mg curcumin by gavage, topical, or both, and tumors were measured daily with digital calipers. Xenograft tumors did not form in one animal per group and were excluded (*n* = 9 per group). Tumor volume (mm^3^) was calculated using the following formula: (0.52 × length^2^ × width). Body weight was measured daily, and mice were monitored for adverse effects from the experiment. Daily oral gavage and tumor volume measurement continued through day 29, at which time tumors were harvested after the mice were anesthetized with isoflurane and sacrificed. *Ex vivo* tumor volume was calculated using the following formula: (4/3*π*0.5 × length × 0.5 × width × 0.5 × height). The study pathologist (FA) measured maximum skin thickness, including the stratum corneum but not the granular layer.

### 2.5. ELISA

Pooled serum from mice (*n* = 3/group) was analyzed by enzyme-linked immunosorbent assay (ELISA, BD Bioscience) according to the manufacturer's instructions, to assess expression of human and murine IL6. Samples were analyzed in duplicate for IL-6 expression with a spectrophotometric plate reader.

### 2.6. Immunohistochemical Analysis of Molecular Markers in Skin Squamous Cell Carcinoma

Tumors harvested on day 29 were embedded in paraffin, sectioned, and H&E stained for confirmation of squamous cell carcinoma presence by our study pathologist (FA). Tumors (*n* = 3 per group) were then stained with phospho-ERK (cell signaling, Thr202/Tyr204; 1 : 600) and phospho-STAT3 (cell signaling, Tyr705; 1 : 200) as previously described [15, 16]. Subcellular localization was determined by immunofluorescence. Paraffin sections of tumors with overlying mouse skin were probed with pERK1/2 and pSTAT3 antibodies (Cell Signaling) followed by an Alexa-546-labeled secondary antibody. 

Human actinic keratosis, skin SCC, and BCC paraffin-embedded blocks were sectioned and stained with phospho-p44/42 MAPK (ERK 1/2) rabbit monoclonal antibody (Thr202/Tyr204, 1 : 600) and phospho-S6 ribosomal protein rabbit monoclonal antibody (Ser235/236, 1 : 100) as previously described [17–19] and read by our study pathologist (FA). Specimens were scored based on the intensity of antibody nuclear and cytoplasmic staining in each slide, with absence of staining scored as a [0], weak or focal staining scored as a [+], and strong staining with a [++]. 

### 2.7. Western Blot Analysis

Soluble proteins extracted from SRB12-p9 cell lysates treated with 0 *μ*M or 20 *μ*M curcumin for 24 hours or xenograft tumors were analyzed by western blot as previously described [19]. Proteins were detected using enhanced chemiluminescence (Amersham Pharmacia Biotech, Piscataway, NJ, USA) and analyzed with ImageQuant TL7.0 (GE Healthcare) software (*n* = 6/group). The following antibodies from cell signaling were used: AKT (1 : 200), phospho-AKT (Ser^473^; 1 : 100), S6 ribosomal protein (1 : 500), phospho-S6 ribosomal protein (Ser^235/236^; 1 : 500), STAT3 (1 : 200), phospho-STAT3 (Tyr705; 1 : 200), 4EBP1 (1 : 200), phospho-4EBP1 (Ser^65^; 1 : 200), ERK1/2 (1 : 200), phospho-ERK1/2 (Thr^202^/Tyr^204^; 1 : 200), and actin (1 : 3500). 

### 2.8. Patient Tissue Samples and Controls

All BCC and SCC tissue samples were obtained from patients recently diagnosed with nonmelanoma skin cancer of the face or neck, after obtaining approval by the institutional review board and obtaining informed consent from all subjects. Patients were treated primarily with surgical resection at Louisiana State University Health Shreveport and the Overton Brooks Veterans Administration Hospital from 2009 to 2011. Formalin-fixed, paraffin-embedded tissue blocks were obtained from 27 BCC tissue samples, 4 Actinic Keratosis (AK) tissue samples, and 17 SCC tissue samples (from 16 SCC patients). Normal human skin samples were surgically obtained from uninvolved adjacent skin in patients undergoing resection for skin cancer. Total of 25 normal (noncancer) skin samples were analyzed in the study. Several 5 *μ*m slides were cut from each tissue block, and one slide was stained with hematoxylin and eosin (H&E) and reviewed by a pathologist to confirm pathologic findings and assess surgical margins. All other slides were used for immunohistochemical staining.

### 2.9. Statistics Applied for the Analysis

Proliferating cell percentages were compared using one-way analysis of variance (ANOVA). One-way ANOVA was also used to determine significant differences in skin thickness and the differences between individual treatment groups. A Tukey's multiple comparison as a *post hoc* test was performed to evaluate differences between treatment groups. Tukey's *post-hoc* testing, Chi-square test for independence, or Fisher's exact probability test was used to determine the ability of pERK and pS6 expression to correlate with cutaneous SCC, differentiate tumor types from normal skin and BCC, and determine if there was a significant difference between pERK and pS6 staining and the different types of histologic cutaneous lesions. Paired *t*-test was used to determine significant difference in biomarker expression by western blot analysis.

## 3. Results

### 3.1. Growth Inhibitory Effects of Curcumin *In Vitro* and *In Vivo*

**Figure 1:**
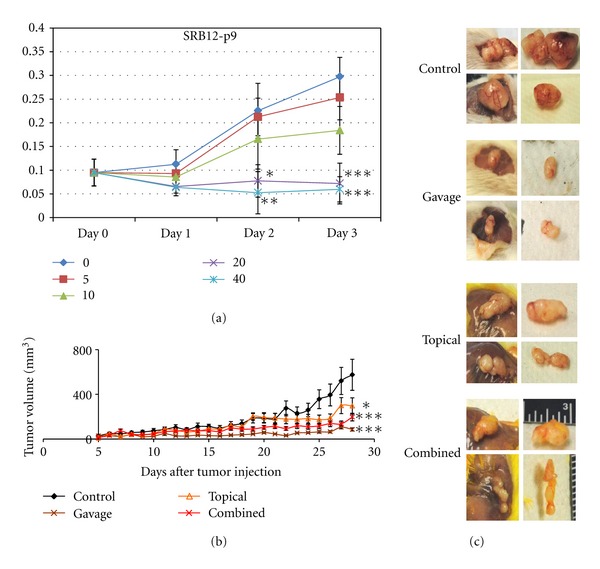
Curcumin inhibits skin SCC cell growth *in vitro* and *in vivo*. (a) Cell proliferation of the aggressive skin cancer cell line SRB12-p9 after treatment with 0–40 *μ*M curcumin. *P < 0.05 versus control group; **P < 0.01 versus control group; ***P < 0.001 versus control group. (b) Mice were pretreated with the indicated dose of curcumin for 3 days prior to injection with 1 × 10^6^ SRB12-p9 tumor cells in the dorsal region (day 0) and continued receiving daily curcumin treatment (9 mice per group, mean tumor volume ± SD). Tukey's *post hoc* test: *P < 0.05 versus control group; ***P < 0.001 versus control group. (c) Representative images of xenograft tumors at harvest and *ex vivo *from the indicated treatment groups.

To determine whether a skin SCC cell line is sensitive to curcumin, a cell proliferation assay was performed on SRB12-p9 SCC cell line. Curcumin's growth inhibitory effects in the aggressive skin cancer cell line (SRB12-p9) were noted as early as day 2 at 20 *μ*M (*P* < 0.05) curcumin compared to control. Curcumin treatment at doses 20 *μ*M and 40 *μ*M was significantly effective in inhibiting the proliferation of SRB12-p9 cells compared to control on days 2 and 3 (*P* < 0.05; Figure 1(a)).

Curcumin appears to inhibit growth compared to control in SRB12-p9 xenograft tumors after tumor cells had a chance to engraft (Figure 1(b)). There was a significant effect for curcumin treatment  (*F*(3, 96) = 11.58, *P* < 0.001) in suppressing growth of the SRB12-p9 xenograft tumors. Tukey's *post hoc* comparisons of the four groups indicate tumor volume from the gavage group (*M* = 44.55, 95% CI [35.77, 53.77]) and the combined group (*M* = 88.81 CI [71.73, 105.89]) was significantly smaller than the control group tumor volume (*M* = 191.35, 95% CI [127.12, 255.59]), *P* < 0.001. The topical group (*M* = 130.66, 95% CI [95.29, 166.04]) tumor volume was also statistically smaller than the control group tumor volume (*P* = 0.02). There was no difference between the gavage group tumor volume and the topical group tumor volume (*P* = 0.19). 

Because invasive tumors could give inaccurate measurements and overlying skin could influence *in vivo* tumor measurements, we also measured tumors *ex vivo* and measured skin thickness (Figure 1(c)). There was a significant effect of curcumin on *ex vivo* tumor volume (*F*(3, 32) = 5.49, *P* = 0.004). Tukey's *post hoc* comparisons of the four groups indicate that the tumor volumes from the gavage group (*M* = 72.06, 95% CI [37.78, 106.35]), topical group (*M* = 195.82, 95% CI [71.59, 320.05]), and combined group (*M* = 152.32, 95% CI [101.048, 203.60]) were significantly smaller than the control (*M* = 416.77, 95% CI [161.48, 672.06]), *P* < 0.001, *P* = 0.006 and 0.02, respectively. There was a significant effect for curcumin treatment on tumor mass (*F*(3,32) = 5.79,  *P* = 0.003), where the gavage group (*M* = 0.043, 95% CI [0.02, 0.07]), topical group (*M* = 0.112, 95% CI [0.041, 0.184]), and combined treatment group tumors (*M* = 0.076, 95% CI [*M* = 0.050, 0.101]) were significantly smaller than that of the control group (*M* = 0.244, 95% CI [0.09, 0.39]) tumors, *P* < 0.001, *P* = 0.02, and 0.003, respectively. There was no difference in skin thickness in mice treated with curcumin by gavage, topical, and combined groups compared to the control group (*P* = 0.73).

### 3.2. Curcumin's Effects on Signaling Pathways

**Figure 2:**
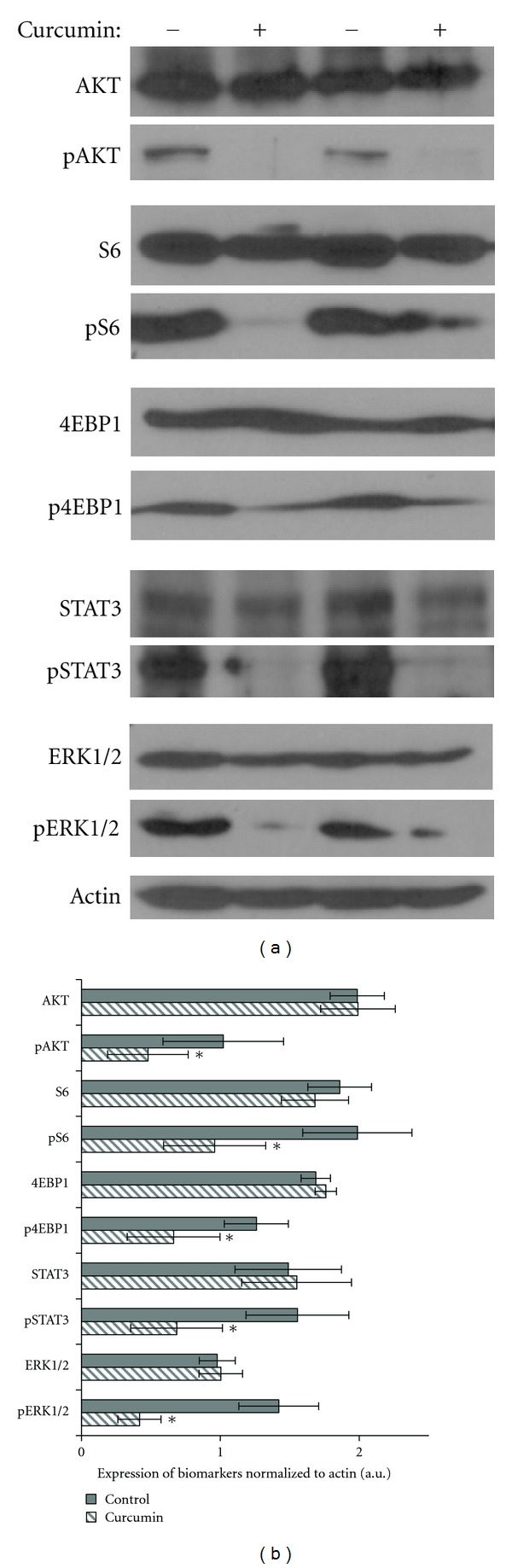
Curcumin's effects on AKT/MTOR and ERK pathways *in vitro*. (a) Western blot of SRB12-p9 tumor cells treated with (+) or without (−) 20 *μ*M curcumin for 24 hours and probed with the indicated antibody. Representative Western blots for two analyzed sets are shown. (b) Band densities of indicated biomarkers (*n* = 6) were quantified using ImageQuant software and normalized to actin protein level. Data presented as Mean ± SE. * Indicates *P* < 0.05 versus vehicle-treated control. A significant inhibition of expression of the following biomarkers was observed: pAKT (*P* = 0.0368); pS6 (*P* = 0.0182); p4EBP1 (*P* = 0.0098); pSTAT3 (*P* < 0.0001); pERK1/2 (*P* = 0.0313). a.u.: arbitrary units.

We next evaluated curcumin's effects on signaling pathways in the aggressive skin cancer cell line (SRB12-p9) *in vitro*. Using a concentration that significantly inhibited cell growth (20 *μ*M), there was significant inhibition of pAKT, pS6, p-4EBP1, pSTAT3, and pERK1/2 (Figure 2). As can be seen in Figure 2 there was about twofold inhibition in the phosphorylation of the aforementioned markers in SRB12-p9 cells after curcumin treatment.

**Figure 3:**
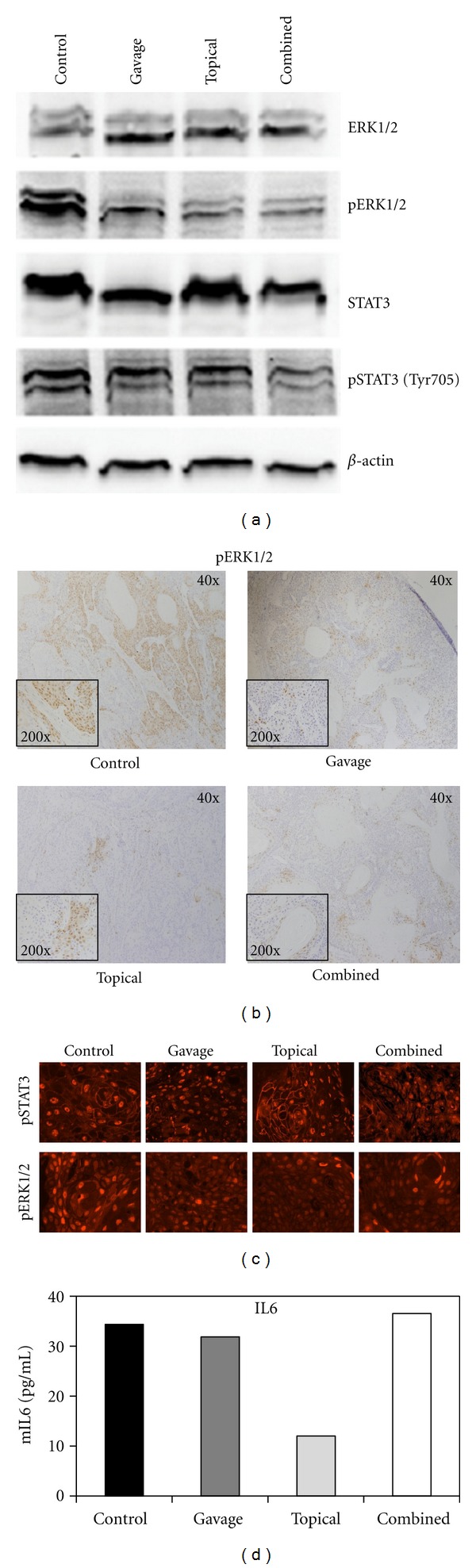
Curcumin's effects on the ERK pathway *in vivo*. (a) Western blot of pooled xenograft tumors (*n* = 6/group) of the indicated antibody. (b) The presence and intensity of pERK staining (brown) in the control group compared to the presence and intensity of pERK staining in the curcumin-treated xenograft tumors. (c) Representative IHC staining of SRB-12 p9 WT cell tumor xenografts. Paraffin sections of tumors were probed with STAT3 phospho-Tyr705 (pSTAT3, top row) or ERK1/2 phospho-Thr202/Tyr204 (pERK, bottom row), followed by an Alexa546-labeled secondary antibody (400x). (d) IL-6 ELISA of pooled mouse serum (*n* = 3/group) in duplicate.

We next evaluated curcumin's effects on signaling pathways in xenograft tumors using western blot analysis (Figure 3). Among the tested biomarkers an inhibition of pERK1/2 was noted in the curcumin-treated groups, whereas inhibition of pSTAT3 was only noted in the combined curcumin group (Figure 3(a)).

As western blot analysis involves homogenization of total tumor tissue, such as stroma and infiltrating host inflammatory cells, we also evaluated curcumin's effects on signaling pathways by immunohistochemistry, which can distinguish nonviable and nontumor components, such as stroma, that are not included in the scoring of the biomarker analyzed. IHC results revealed strong positive pERK staining throughout tumors in the control group and weaker, focal staining in the curcumin-treated tumors (Figure 3(b)). Immunofluorescence confirmed curcumin's effects on pERK and a shift in the subcellular localization of the activated state of STAT3 in the topical group compared to the control group (Figure 3(c)). Curcumin is known for its anti-inflammatory effects. Therefore, we evaluated its effects on the inflammatory marker IL6 in all curcumin treatment groups using pooled serum samples. The levels of soluble IL6 were the lowest in the topical curcumin group, while curcumin did not affect IL6 levels in the gavage or combined groups (Figure 3(d)).

### 3.3. Patient Characteristics

Patient demographics and clinical characteristics are summarized in Table 1. Tissue samples from 46 male patients and 4 female patients were analyzed. Age ranged from 39 to 93 with a mean age of 66 ± 14 years. There was no difference in age between the groups by ANOVA (*F* = 1.272, *P* = 0.29). The large majority of patients were white, except for one African American patient with albinism. Nonmelanoma skin cancers analyzed were excised from the external nasal skin (14), cheeks (14), ears (9), scalp and forehead (13), neck, chin, and lip (6). No skin site was overrepresented in analysis.

### 3.4. IHC Analysis of Patient Tissues

**Figure 4:**
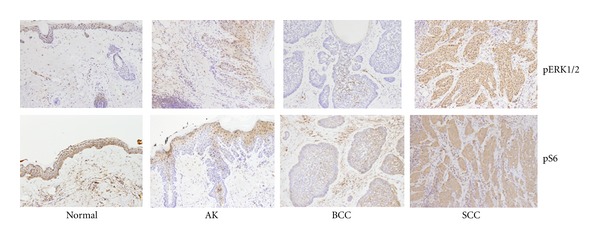
IHC analysis of pS6 and pERK expression in patients with negative (blue) staining and strong positive (brown) staining of tumor cells with pERK and pS6. Normal patient skin samples with minimal background staining and normal appearing cells. Representative actinic keratosis (AK) patient samples showing weak, cytoplasmic staining. Representative BCC patient samples with negative (blue) staining and few scattered positive (brown) staining of tumor cells. Representative SCC patient samples with strong positive (brown) nuclear and cytoplasmic staining with pERK and pS6. Note that the stroma stains positive (brown) in BCC, whereas the tumor stains negative (blue).

The presence and intensity of pERK and pS6 staining in all SCC, BCC, and normal tissue samples were compared (Table 2). All SCC specimens (*n* = 17, 100%) stained positive for phosphorylated ERK, while only 10 of 27 (37%) BCC samples stained positive. Although all the normal skin samples stained weakly positive (grade 1+) for activated pERK in the stroma, palisading cells, and epithelium (*n* = 24, 100%), significantly more SCC specimens showed strong staining with pERK (grade 2+) than normal skin (*P* = 0.0028, Table 2 and Figure 4). However, the majority of BCC specimens (17/27, 63%) showed no pERK staining (*P* < 0.0001 compared to normal skin). 

Most specimens containing SCC (*n* = 13, 81%) and BCC (*n* = 16; 64%) showed strong staining (grade 2+) for activated pS6, while all the analyzed normal skin specimens (*n* = 8; 100%) demonstrated negative pS6 staining. Tumor specimens expressed significantly more activated pS6 than normal skin samples (1+ score and above; *P* < 0.0001; Figure 4). Skin cancer type significantly predicted intensity of pERK staining, as SCC tumors stained more intensely for pERK than the background stroma in normal skin and BCC tumor cells (*P* < 0.0001; Figure 4). When pERK expression was analyzed and compared to other demographic factors, the variance in pERK expression scores correlated significantly with tumor type, *R* ^2^ = 0.25, *P* = 0.0007. Patient age (*P* = 0.85) and gender (*P* = 0.35) did not explain the variance in pERK staining.

## 4. Discussion

Identifying consistent intracellular biomarkers at which a potential chemopreventive may act is essential prior to initiating clinical trials. As curcumin acts on many different biomolecular targets in a variety of different cell types it is important to determine if curcumin directly affects either a few major downstream biomarkers or a multiplicity of downstream targets which may serve to explain curcumin's varying effects in different cell types. Aberrant signaling through the epidermal growth factor receptor (EGFR) plays a major role in cutaneous skin cancer progression. EGFR inhibitors have been used for SCC therapy to downregulate aberrant EGFR signaling with little change in overall survival [20], possibly due to compensating mutations downstream of EGFR. One of these signaling pathways is PI3 K/AKT that plays a role in skin carcinogenesis and in chemotherapy resistance [21]. 

Activated Ras/Raf signaling has also been implicated in a small percentage of SCC [22] and can lead to activation of the MAPK pathway. Ras/Raf gain-of-function can occur through activation of ERK1 and ERK2, which are constitutively active in 70% of malignant melanoma due to RAS or BRAF activating mutations [2]. Activated ERK1/2 is rarely seen in normal skin specimens but is shown in all cases of SCC with a positive association with the degree of malignancy and proliferative activity of SCC [23]. In this study, Zhang et al. looked at 10 well-differentiated and 10 poorly differentiated skin SCC cases. Another study looked at activated ERK in 101 human head and neck squamous carcinoma specimens [24]. Therefore, inhibiting ERK may be a promising approach in targeted cutaneous skin SCC therapy. Having previously determined curcumin's growth inhibitory effects in skin SCC [11], we sought to determine whether these effects were similar to our observations in upper aerodigestive head and neck SCC (HNSCC) where curcumin inhibited the AKT/MTOR pathway through rapid curcumin-dependent inhibition of MTOR's downstream target pS6 and 4EBP1 phoshorylation [18].

In this study we found significant and complete inhibition of SRB12-p9 cell proliferation after treatment with curcumin at a dose 20 *μ*M or higher (Figure 1(a)) suggesting a highly potent anticarcinogenic effect of curcumin in skin cancer. Additionally, we found that the inhibitory effect of curcumin on skin cancer proliferation was associated with inhibition of AKT/mTOR and ERK signaling (Figure 2).

In our *in vivo* study, curcumin paste was formulated to penetrate human skin epidermis and dermis. However, given the thin nature of mouse skin, topical curcumin penetration was much greater such that curcumin possibly did not remain in the epidermis for a prolonged period, leading to prolonged contact with the cancer cells. The irritant nature of the cream caused the skin overlying the tumor to thicken, although this was not statistically significantly different from control (*P* = 0.73). The SRB12-p9 cell line is invasive in this model [25], producing inaccurate tumor caliper measurements due to the inability to account for the portion of the tumor that invaded into the abdominal wall. Therefore, the *ex vivo* tumor weight provided a more accurate tumor size endpoint. In human skin, SCC emerges directly from the epidermal layer, unlike in our xenograft model, where tumor is encapsulated under the epidermis. We therefore anticipate a more pronounced tumor-suppressive effect of topical curcumin in humans.

The SRB12-p9 xenograft cells were more sensitive to curcumin-induced cell death and apoptosis than the surrounding normal mouse skin and grew at a much slower rate in the presence of curcumin, whether topical or systemic, compared to control. IL6 plays a central role in regulating the inflammatory response [25]. Because IL-6 may contribute to angiogenesis and metastasis [26], inhibition of IL-6 with topical curcumin suggests a mechanism of chemoprevention. Although curcumin has previously been shown to inhibit IL-6 in HNSCC cell lines [27], this is the first skin cancer model investigating curcumin's inhibition of systemic IL-6. The present study demonstrates that topical curcumin reduces skin SCC tumor growth, and this effect might be explained, by the inhibition of IL-6. 

In this study we demonstrated significant inhibition of several biomarkers of the AKT/mTOR pathway as well as STAT3 and ERK1/2 in SRB12-p9 cells after treatment with 20 *μ*M of curcumin. In our *in vivo* experimentation, we observed inhibition of pERK in the curcumin-treated tumors and inhibition of pSTAT3 in the combined curcumin group. However, tumor heterogeneity and degree of dysplasia can often confound immunohistochemistry results, depending on where in the lesion the biopsy was taken. Therefore, it is important to develop serum biomarkers that can be obtained with a simple blood draw. As curcumin is a well-known anti-inflammatory agent, we measured its effects on pooled serum of treated mice and noted a decrease in IL-6 in the topical group compared to the control group. We observed that systemic curcumin did not cause a decrease in serum IL-6 levels. However, only three mice in each group were analyzed, and it is possible that statistically significant differences in IL6 levels could be detected upon analysis of greater numbers of mice in the topical and combined curcumin-treated groups compared to control mice. 

As curcumin slowed progression of aggressive skin SCC xenografts and inhibited pERK expression, the ERK pathway may prove to be a key biomarker in developing topical pharmaceutical agents that prevent skin SCC tumor growth or recurrence. We observed that the overall reduction in pERK staining in the curcumin-treated tumors was not cell autonomous but rather manifested as an expansion in areas of very low or no expression, such that focal regions of intense staining remained. Alternatively, control tumors had smaller regions of low staining and a higher number of intensely staining areas. This indicates that a global reduction of pERK staining was achieved with curcumin treatment, rather than a complete shutdown. [23] confirmed that phosphorylated ERK is overexpressed in patient skin SCC in a Caucasian population, which further supports our findings and suggests that pERK may be a useful chemoprevention biomarker.

Chronic inflammation is linked to both cancer and angiogenesis. The anti-inflammatory properties of curcumin may contribute to its potential as an effective chemopreventive agent. However, curcumin's systemic anti-inflammatory effects (reduced serum IL-6 levels) were more pronounced in topical curcumin group compared to gavage. Given these findings, it was unexpected that tumor growth was inhibited more effectively in the gavage group than in the topical group. However, there was no statistically significant difference in tumor volume between the two treatment groups. Despite this data, we speculate that local anti-inflammatory activity of topically applied curcumin contributes significantly to its chemopreventive activity, circumventing its poor systemic bioavailability. 

As curcumin continues to be explored as a chemopreventive and therapeutic agent for skin cancer treatment, establishing defined biomarkers upon which curcumin acts to inhibit tumorigenesis is essential. The ERK pathway is an important protein kinase signaling cascade involved in cellular proliferation and is activated in carcinogenesis. In this study, activated pERK expression significantly increased in SCC compared to the less aggressive BCC and AK. As curcumin has been shown to inhibit activated ERKs in carcinogenesis, the present data suggests that components of the ERK pathway may prove to be key biomarkers for curcumin chemopreventive efficacy in cutaneous SCC.

## Figures and Tables

**Table 1:** Clinical and demographic patient characteristics.

	Total	Normal*	AK	SCC	BCC	*P* value
Gender**						
Male	46	21	4	14	25	0.77***
Female	4	4	0	2	2	
Race						
White	49	24	4	15	27	0.57***
African American	1	1	0	1	0	
Age						
<60	17	13	1	7	7	0.44***
60–70	14	7	1	3	9	
>70	19	5	2	6	11	
Skin site						
Nose	14	6	2	2	8	0.71***
Cheeks	14	7	0	8	6	
Ear	9	5	1	1	5	
Scalp and forehead	13	5	1	5	5	
Other	7	4	0	2	3	

*Normal skin samples were surgically obtained from uninvolved adjacent skin in patients undergoing resection for skin cancer.

**Some patients had more than one type of cancer and are counted in both groups.

***No significant difference in number of males and females, race, age, or skin site distribution per group by Fisher's exact test.

**Table 2:** Summary of pERK and pS6 IHC staining in normal (noncancer), AK, BCC, and SCC skin samples.

	[0]	[1+]	[2+]	*P* value*	Total
	pERK staining				

Normal Skin	0	24	0		24
AK	0	4	0	1.0000	4
BCC	17	5	5	<0.0001	27
SCC	0	11	6	0.0028	17

	pS6 staining				

Normal Skin	8	0	0		8
AK	2	0	2	0.0909	4
BCC	5	4	16	<0.0001	25
SCC	1	2	13	<0.0001	16

*Compared to normal skin by Fisher's exact test. *P* values for overall comparison are shown. See text for a subset analysis.

## References

[1] Alam M, Ratner D (2001). Cutaneous squamous-cell carcinoma. *The New England Journal of Medicine*.

[2] Green CL, Khavari PA (2004). Targets for molecular therapy of skin cancer. *Seminars in Cancer Biology*.

[3] Rowe DE, Carroll RJ, Day CL (1992). Prognostic factors for local recurrence, metastasis, and survival rates in squamous cell carcinoma of the skin, ear, and lip: implications for treatment modality selection. *Journal of the American Academy of Dermatology*.

[4] de Lima Vazquez V, Sachetto T, Perpetuo NM, Carvalho AL (2008). Prognostic factors for lymph node metastasis from advanced squamous cell carcinoma of the skin of the trunk and extremities. *World Journal of Surgical Oncology*.

[5] Joseph MG, Zulueta WP, Kennedy PJ (1992). Squamous cell carcinoma of the skin of the trunk and limbs: the incidence of metastases and their outcome. *Australian and New Zealand Journal of Surgery*.

[6] Kwa RE, Campana K, Moy RL (1992). Biology of cutaneous squamous cell carcinoma. *Journal of the American Academy of Dermatology*.

[7] Kraus DH, Carew JF, Horrison LB (1998). Regional lymph node metastasis from cutaneous squamous cell carcinoma. *Archives of Otolaryngology—Head and Neck Surgery*.

[8] Kuttan R, Sudheeran PC, Josph CD (1987). Turmeric and curcumin as topical agents in cancer therapy. *Tumori*.

[9] Hsu CH, Cheng AL (2007). Clinical studies with curcumin. *Advances in Experimental Medicine and Biology*.

[10] Dujic J, Kippenberger S, Ramirez-Bosca A (2009). Curcumin in combination with visible light inhibits tumor growth in a xenograft tumor model. *International Journal of Cancer*.

[11] Phillips JM, Clark C, Herman-Ferdinandez L (2011). Curcumin inhibits skin squamous cell carcinoma tumor growth *in vivo*. *Otolaryngology—Head and Neck Surgery*.

[12] Hua WF, Fu YS, Liao YJ (2010). Curcumin induces down-regulation of EZH2 expression through the MAPK pathway in MDA-MB-435 human breast cancer cells. *European Journal of Pharmacology*.

[13] Vourlekis JS, Szabo E (2003). Predicting success in cancer prevention trials. *Journal of the National Cancer Institute*.

[14] Clifford JL, Yang X, Walch E, Wang M, Lippman SM (2003). Dominant negative signal transducer and activator of transcription 2 (STAT2) protein: stable expression blocks interferon alpha action in skin squamous cell carcinoma cells. *Molecular Cancer Therapeutics*.

[15] Nathan CAO, Amirghahari N, Abreo F (2004). Overexpressed eIF4E is functionally active in surgical margins of head and neck cancer patients via activation of the Akt/mammalian target of rapamycin pathway. *Clinical Cancer Research*.

[16] Kleiner-Hancock H, Shi R, Remeika A (2010). Effects of ATRA combined with citrus and ginger-derived compounds in human SCC xenografts. *BMC Cancer*.

[17] Syed Z, Cheepala SB, Gill JN (2009). All-trans retinoic acid suppresses Stat3 signaling during skin carcinogenesis. *Cancer Prevention Research*.

[18] Clark CA, McEachern MD, Shah SH (2010). Curcumin inhibits carcinogen and nicotine-induced mammalian target of rapamycin pathway activation in head and neck squamous cell carcinoma. *Cancer Prevention Research*.

[19] Yin W, Cheepala S, Roberts JN, Syson-Chan K, DiGiovanni J, Clifford JL (2006). Active Stat3 is required for survival of human squamous cell carcinoma cells in serum-free conditions. *Molecular Cancer*.

[20] Wheeler DL, Dunn EF, Harari PM (2010). Understanding resistance to EGFR inhibitors-impact on future treatment strategies. *Nature Reviews Clinical Oncology*.

[21] Claerhout S, Verschooten L, Van Kelst S (2010). Concomitant inhibition of AKT and autophagy is required for efficient cisplatin-induced apoptosis of metastatic skin carcinoma. *International Journal of Cancer*.

[22] Pierceall WE, Goldberg LH, Tainsky MA, Mukhopadhyay T, Ananthaswamy HN (1991). ras Gene mutation and amplification in human nonmelanoma skin cancers. *Molecular Carcinogenesis*.

[23] Zhang X, Makino T, Muchemwa FC (2007). Activation of the extracellular signal-regulated kinases signaling pathway in squamous cell carcinoma of the skin. *Bioscience Trends*.

[24] Albanell J, Codony-Servat J, Rojo F (2001). Activated extracellular signal-regulated kinases: association with epidermal growth factor receptor/transforming growth factor *α* expression in head and neck squamous carcinoma and inhibition by anti-epidermal growth factor receptor treatments. *Cancer Research*.

[25] Rose-John S, Scheller J, Elson G, Jones SA (2006). Interleukin-6 biology is coordinated by membrane-bound and soluble receptors: role in inflammation and cancer. *Journal of Leukocyte Biology*.

[26] Benoy I, Salgado R, Colpaert C, Weytjens R, Vermeulen PB, Dirix LY (2002). Serum interleukin 6, plasma VEGF, serum VEGF, and VEGF platelet load in breast cancer patients. *Clinical Breast Cancer*.

[27] Cohen AN, Veena MS, Srivatsan ES, Wang MB (2009). Suppression of interleukin 6 and 8 production in head and neck cancer cells with curcumin via inhibition of I*κβ* kinase. *Archives of Otolaryngology—Head and Neck Surgery*.

